# Machine Learning Potentials for Inorganic and Hybrid Lead Halide Perovskites: From Phase Stability to Defects and Interfaces

**DOI:** 10.1021/acsami.5c24460

**Published:** 2026-03-19

**Authors:** Tieyuan Bian, Wenjia Zhu, Qiong Lei, Jun Yin

**Affiliations:** † Department of Applied Physics, 26680 The Hong Kong Polytechnic University, Hung Hom, Kowloon, Hong Kong 999077, China; ‡ Macao Institute of Materials Science and Engineering (MIMSE), Faculty of Innovation Engineering, 58816 Macau University of Science and Technology, Taipa, Macao, 999078, China

**Keywords:** *machine learning potentials*, *lead halide perovskites*, *molecular dynamics*, *phase transition*, *ion migration*, *point defect and grain boundary*

## Abstract

Inorganic and hybrid lead halide perovskites are among the most promising candidates for next-generation optoelectronic devices. However, their further development is critically constrained by stability issues, including phase degradation, migration, and interfacial reactions. Overcoming these challenges requires a detailed understanding of the underlying atomic-scale mechanisms. Density functional theory (DFT) and ab initio molecular dynamics (AIMD) offer essential electronic and structural insights, but their high computational cost limits simulations to relatively small systems and short time scales, making it difficult to capture long-term evolution. Classical MD can access larger length and time scales but often lacks accurate and transferable force fields for lead halide perovskite systems. Machine learning potentials (MLPs) have emerged as an alternative solution to bridge this gap, enabling large-scale, long-time-scale MD simulations with near-DFT accuracy at a fraction of the computational cost. In this perspective, we review recent applications of MLPs to inorganic and hybrid lead halide perovskites, including phase behavior, ion migration, and perovskite interfaces. We further discuss current challenges related to model efficiency and transferability and outline future opportunities for deploying MLPs to tackle outstanding questions in perovskite stability, degradation, and device-relevant operation.

## Graphical abstract

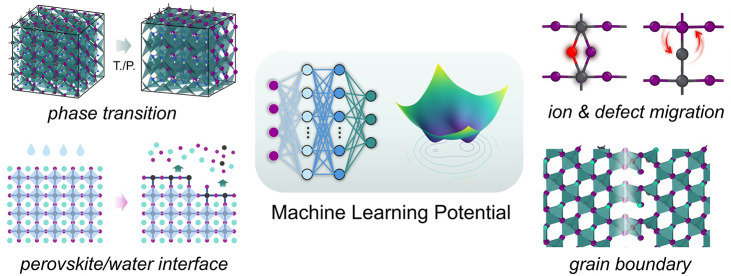

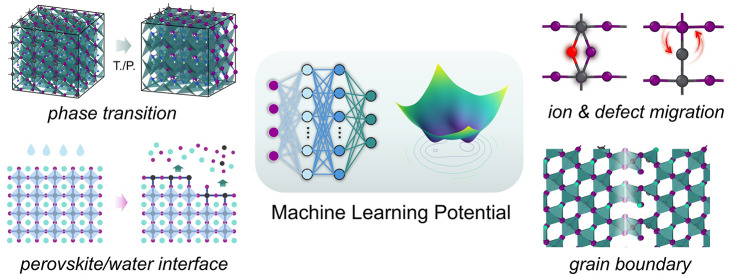

## 1 Introduction

Inorganic and hybrid lead halide perovskites have emerged as a highly promising class of materials for optoelectronic and spintronic applications.^1–5^ All-inorganic systems such as the CsPbX_3_ (X = Cl, Br, I) and their hybrid counterparts, including methylammonium lead halides (MAPbX_3_) and formamidinium lead halides (FAPbX_3_), have attracted widespread attention owing to their exceptional physical properties, including high absorption coefficients, long charge carrier lifetimes, high mobilities, and tunable bandgaps.^6–10^ These properties make them ideal candidates for high-efficiency photovoltaics,^11–13^ light-emitting diodes,^14–16^ X-ray scintillators and detectors,^17–19^ and spintronic devices.^20–22^ However, large-scale commercialization of these materials is currently hindered by critical stability issues. The primary challenges include phase instability and the inherent vulnerability of the soft lattice, which renders these materials highly susceptible to environmental factors such as moisture and heat.^23,24^ In addition, intrinsic instabilities driven by ion migration and defect accumulation severely compromise long-term device reliability.^25,26^ Addressing these problems requires an atomistic understanding of structure–property relationships and degradation pathways. Although advanced experimental techniques—ranging from atomic force microscopy (AFM)^27^ and transmission electron microscopy (TEM)^28^ to ultrafast spectroscopy^29^—have been extensively employed to characterize these systems, obtaining comprehensive atomic-scale information, particularly on intrinsic properties and ultrafast transient phenomena, remains extremely challenging. Consequently, theoretical studies that provide complementary information are urgently needed to bridge this gap.

Among available theoretical approaches, density functional theory (DFT) and molecular dynamics (MD) have become the main approaches for investigating perovskite materials.^30–34^ For systems containing several hundred atoms, DFT enables accurate calculations of electronic structures and energetics. Ab initio molecular dynamics (AIMD) extends DFT to the simulation of finite-temperature dynamics, but it is usually limited to time scales below 100 ps. These computational methods have provided crucial insights into the electronic structure, defect behavior, and surface chemistry of perovskites.^35–37^ However, their substantial computational cost restricts their applicability to relatively small systems and short time scales, making it difficult to capture the long-term structural evolution of perovskites. Classical MD simulations with empirical or semiempirical interatomic potentials offer a practical alternative for investigating larger length and time scales. Such simulations routinely access systems with more than 10,000 atoms and time scales beyond 1 ns, making them well suited for investigating processes with long-time correlations, such as phase transitions, domain dynamics, and grain boundary (GB) evolution. Yet these classical potentials are typically fitted to a limited set of experimental or ab initio data to reproduce specific properties, which constrains their transferability and overall accuracy. As a result, conventional MD often struggles to describe chemically complex scenarios, such as solid–liquid interfaces, where the local chemical bonding environment evolves significantly in time.

Modern machine learning potentials (MLPs) have been developed to overcome the respective limitations of DFT/AIMD and classical MD. Figure 1 schematically illustrates the construction and applications of MLPs. In brief, a diverse set of atomic configurations is generated, and their energies and forces are calculated using first-principles methods. These data are then used to train machine learning algorithms that approximate high-dimensional potential energy surface (PES) suitable for subsequent MD simulations.^38–45^ MLP-based MD enables simulations of systems comprising tens of thousands of atoms on time scales of up to several tens of nanoseconds, thereby greatly expanding the accessible phase space for perovskite systems. Properly trained MLPs can achieve accuracy comparable to AIMD, with typical errors of ∼5–10 meV/atom in energies and ∼50–100 meV/Å in forces. However, the adequacy of these metrics is strongly property dependent, because the relevant energy scales vary widely across perovskite phenomena. For phase transitions, the free energy differences between competing phases are often subtle (often <10 meV/atom). In this regime, accurate predictions of transition temperatures generally require highly accurate relative energetics to remain within experimentally meaningful uncertainty (e.g., ± 30 K). For ion migration, where kinetics follow Arrhenius dependence, keeping errors in migration barriers below ∼0.05 eV is essential; otherwise, even modest barrier errors can translate into orders-of-magnitude deviations in predicted diffusivities. For defect formation, although the absolute energy scale is typically larger than for phase transitions, accurate defect energetics still hinge on faithful local structural relaxation; small structural inaccuracies can propagate into substantial errors in predicted defect concentrations. Accordingly, global error metrics should always be evaluated against the specific energy scales of the physical process being simulated.

**Figure 1:**
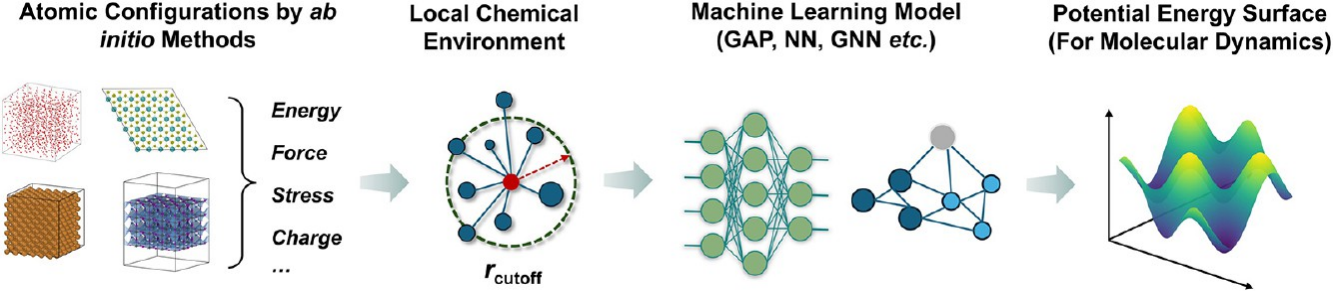
Construction of machine learning potentials (MLPs). Ab initio calculations provide reference data (energies, forces, stresses, charges) for diverse atomic configurations. Local chemical environments are mapped to descriptors and used to train an ML model (e.g., Gaussian approximation, neural networks, graph neural networks), which yields a potential energy surface suitable for molecular dynamics simulations.

Since the first MLP was reported in 1995,^46^ these methods have evolved from proof-of-concept demonstrations to widely used tools in computational materials modeling. Their impact is particularly evident in the study of inorganic and hybrid lead halide perovskites, where MLPs are now recognized as powerful and reliable techniques for bridging electronic-structure accuracy and realistic simulation scales. Given the wide scope of perovskite, a complete review of all ML applications in this field is beyond the scope of this work. Instead, this perspective focuses specifically on the use of MLPs to obtain atomic-level insights into perovskite phase stability, defect chemistry, and interfacial phenomena. Other important ML applications in perovskite research, such as high-throughput screening of new structures and organic cations or data-driven optimization of fabrication processes, have already been extensively explored and are therefore not covered here, despite their high relevance and rapid growth.^47–51^ The perspective is organized as follows. We first provide a concise overview of MLP methodologies as applied to perovskite systems, followed by a summary of key achievements in elucidating fundamental physical and chemical processes. We then highlight critical challenges in perovskite research that have been addressed using MLP-based approaches. Finally, we offer a forward-looking perspective on emerging opportunities and future directions for machine learning potentials in the study of inorganic and hybrid lead halide perovskites.

## 2 Machine Learning Potentials

In recent years, the development of MLPs has advanced rapidly, establishing them as a key tool for atomistic simulations in materials science, especially for perovskite materials. Many different MLP frameworks have been proposed, each with distinct strengths and limitations. In this perspective, which focuses on applications of MLPs to inorganic and hybrid lead halide perovskites, we do not attempt to provide an exhaustive methodological review. Instead, we offer a comprehensive overview of the field and group current models into four broad classes based on their descriptors and symmetry treatment: local descriptor-based models, invariant message-passing neural networks (MPNNs), equivariant MPNNs, and universal atomic models, as summarized in Figure 2. Readers interested in the underlying theory, descriptors, and training strategies of MLPs are referred to several reviews that discuss these aspects in detail, including those by Behler, Parrinello, and co-workers.^40,41,43,44,52–58^

**Figure 2:**
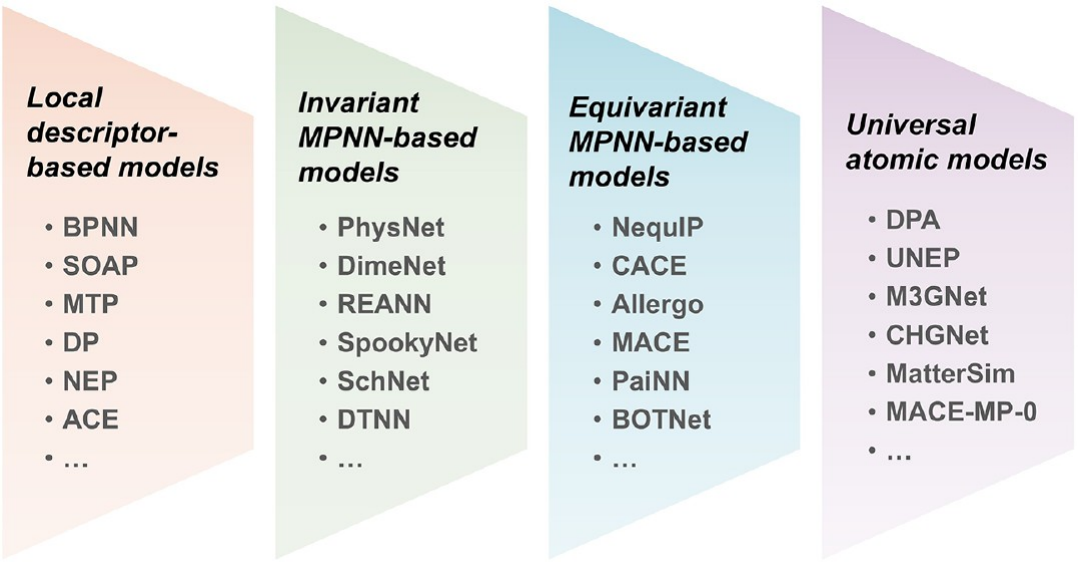
Evolution of representative atomistic MLP models. These models are broadly categorized into four types: local descriptor-based models, invariant MPNN-based models, equivariant MPNN-based models, and universal potential models.

The first and most established class of MLPs represents each atom by a descriptor of its local environment within a finite cutoff radius. These fixed-length, symmetry-preserving descriptors are then passed to a regression model, typically a neural network, to predict atomic energies and forces. For lead halide perovskites, two methods have been used most widely because they combine high accuracy with very low computational cost: the deep potential (DP) method and the neuroevolution potential (NEP).^59–64^ The DP model M can be written in a general form as

$$y_{i}=M\left(x_{i},{\left\{x_{i}\right\}}_{j\in n\left(i\right)};\theta \right)=F\left(D\left(x_{i},{\left\{x_{i}\right\}}_{j\in n\left(i\right)};\theta_{d}\right);\theta_{f}\right)$$

Here, *y*_*i*_ represents the target property to be predicted. The function F corresponds to the fitting network, while *D* is the descriptor function. The state of atom *i* is defined by *x*=(*r*_*i*_,α_*i*_), comprising its Cartesian coordinates *r*_*i*_ and chemical species α_*i*_. The index set *n*(*i*) identifies the neighbors of atom *i* located within a specified cutoff radius. The complete set of trainable parameters is represented by θ = {θ_d_,θ_f_}, which is composed of the parameters for the descriptor (θ_d_, if applicable) and the fitting network (θ_f_). DeePMD-kit supports several types of atomic descriptors, including the local-frame descriptor, the two- and three-body embedding DeepPot-SE descriptor *se_e2*, the attention-based descriptor *se_atten*, and hybrid descriptors that combine multiple schemes. In practice, these descriptors can capture the complex structural and dynamical behavior of lead halide perovskites, including surfaces and interfaces in contact with liquid environments.

The NEP framework, often employed via the GPUMD package, distinguishes itself by using an evolutionary algorithm rather than standard backpropagation for optimization. This approach is particularly robust for finding global minima in the loss function landscape. In NEP, the total potential energy *U* of a system is written as $U=\sum_{i}U_{i}$ , where the site energy *U*_*i*_ of atom *i* is *U*_*i*_ = *U*_*i*_({*r*_*ij*_}). *r*_*ij*_ is defined as the relative position from atom *i* to atom *j*. The local environment is described using a set of radial descriptors *q*_*n*_^*i*^ and angular descriptors *q*_*nl*_^*i*^ (*n* ≥ 0 and *l* ≥ 1):

$$q_{n}^{i}=\underset{j\neq i}{\sum }g_{n}\left(r_{ij}\right)$$

$$q_{nl}^{i}=\sum_{j\neq 1}\sum_{k\neq i}g_{n}\left(r_{ij}\right)g_{n}\left(r_{ik}\right)P_{l}\left({\cos\theta }_{ijk}\right)$$

where *P*_*l*_(cosθ_*ijk*_) is the Legendre polynomial of order l, θ_*ijk*_ being the angle formed by the *ij* and *ik* bonds. The functions *g*_*n*_(*r*_*ij*_) and *g*_*n*_(*r*_*ik*_) are radial functions. These descriptors are fed into a feed-forward neural network with a single hidden layer. The simplified architecture of NEP, together with CUDA-optimized molecular dynamics algorithms, enables exceptionally high computational speeds, making it possible to simulate systems with millions of atoms and thus to study phase transitions and large-scale domain dynamics in perovskites. In parallel, specialized architectures such as DefiNet have been introduced to accelerate defect modeling, achieving millisecond-level structure prediction speeds through a noniterative, defect-informed framework.^65^

However, the reliability of these local descriptor-based models is fundamentally constrained by the quality and coverage of the training data. For soft materials like lead halide perovskites, characterized by strong anharmonicity and a complex phase space, standard AIMD simulations performed only under ambient conditions rarely provide sufficiently diverse sampling of the relevant configuration space. To overcome this limitation, a range of active learning strategies have been developed to expand training data sets and improve phase-space coverage. In perovskite research, these strategies are commonly grouped into two broad classes:

(i) Query-by-Committee (QBC). In this approach, an ensemble of models is trained in parallel, and the dispersion of their predictions is used as an uncertainty indicator for a candidate configuration. Structures associated with large model disagreement are flagged for additional first-principles labeling (typically DFT). This strategy underpins the widely used DP-GEN workflow for constructing DP models.
(ii) Enhanced Sampling. Exploration of the potential energy surface is accelerated by incorporating techniques such as metadynamics, umbrella sampling, or MD at elevated temperatures/pressures, enabling the system to escape local minima and access otherwise rare configurations. Such approaches are frequently employed in the development of NEP models.

Other schemes, such as on-the-fly learning and descriptor-distance-based selection, also offer effective alternatives. By directing expensive DFT calculations toward only the most informative configurations, active learning promotes training sets that better span the physically relevant phase space and reduces the likelihood of extrapolation during the production simulations. Importantly, it mitigates the risk of failure when applying models trained near room temperature to high-temperature phases. Through the iterative exploration and labeling, the resulting potentials can attain the transferability needed to describe phase transitions and defect dynamics over temperature windows of hundreds of kelvins, as discussed in the following sections.

While local descriptor-based methods are highly efficient, their reliance on a fixed cutoff radius can make it challenging to capture long-range interactions and subtle nonlocal charge transfer effects. Invariant MPNNs address this limitation by representing the atomic system as a graph, where atoms are nodes and neighbor relationships are edges. Each atom carries a feature vector that is iteratively updated layer by layer. At each step, the feature of an atom is refined by combining its current state with information collected from its neighbors. In invariant MPNNs such as SchNet^66^ and PhysNet,^67^ these messages are constructed from scalar quantities (e.g., interatomic distances and bond angles) that do not change under rotations of the structure. As a result, the learned atomic representations and the predicted energies are strictly rotationally invariant. Because information is propagated over several message-passing steps, these models can effectively encode structural information beyond the immediate local environment.

A more recent and powerful evolution of this idea is the class of equivariant MPNNs. In contrast to invariant models, which reduce geometric information to scalar features at an early stage, equivariant architecture tracks vector and tensor quantities throughout the network. Their internal features are constructed to transform in a well-defined way under rotations of the atomic configuration: energies remain invariant, while forces and other vector properties rotate consistently with the structure. Architectures such as NequIP,^68^ Allegro,^69^ and MACE^70^ achieve this by using symmetry-adapted basis functions, for example, spherical-harmonic-like features, to encode atomic environments and by combining them through symmetry-preserving tensor operations. This explicit treatment of orientations and angular correlations allows equivariant models to extract more physical information from each training configuration, leading to higher data efficiency than purely invariant networks.

Beyond these system-specific MLP architectures, there is a growing effort to develop universal interatomic potentials that can cover a wide range of chemistry and materials classes. These models are trained on massive and heterogeneous data sets containing millions of atomic configurations, such as Omat24,^71^ Alex2D,^72^ OC22,^73^ and Materials Project (MP) trajectory data sets.^74^ Representative examples include DPA,^75,76^ MACE-MP-0,^77^ CHGNet,^74^ M3GNet,^78^ and NEP89,^79^ which serve as general-purpose potentials capable of handling large parts of the periodic table without task-specific training. They typically build on scalable architectures, often based on equivariant MPNNs or transformer-like designs, to fully exploit the depth of available data. However, directly deploying these universal models in molecular dynamics simulations presents challenges. First, their large parameter counts, symmetry-preserving operations, and substantial memory overhead make inference computationally expensive, limiting the accessible system sizes and simulation time scales. Second, although fine-tuning on perovskite-specific data sets can improve task-level accuracy, it also carries the risk of catastrophic forgetting, whereby the model sacrifices its broad chemical generalizability. Consequently, knowledge distillation using the universal model as a teacher model to label data for a faster student model offers a promising solution. By transferring the high-quality representations of foundation models into lightweight architectures, distillation can retain much of the teacher’s accuracy while achieving the high inference speed needed for routine, large-scale perovskite simulations.

Despite the rapidly expanding landscape of MLP frameworks, studies on inorganic and hybrid lead halide perovskites are still dominated by local descriptor-based models, particularly DP and NEP. Their widespread adoption is largely pragmatic, reflecting mature software ecosystems, very high computational speed, and demonstrated scalability to the large system sizes required for realistic perovskite simulations. By contrast, MPNNs offer higher data efficiency and can capture more complex and long-range interactions. Increasing evidence suggests that nonlocal charge transfer and long-range electrostatic interactions are central to perovskite behaviors that local descriptors with fixed cutoffs often struggle to capture. This limitation becomes particularly evident in processes such as field-driven ion migration and photoinduced segregation, where relevant interactions extend well beyond the local atomic environment. Accordingly, we anticipate a gradual shift toward architecture that can explicitly account for long-range effects, including MPNNs or local descriptor-based models augmented with physically motivated variables (e.g., environment-dependent charges and dipoles). Such a transition is especially important for heterogeneous interfaces, where constructing sufficiently large and diverse training data sets is often computationally prohibitive. In these data-scarce settings, the superior data efficiency of graph-based models can offer a distinct advantage. As MPNN-based potentials become more accessible and better optimized, we expect graph-based potentials to see substantially broader adoption in the coming years.

## 3 Phase Transition and Stability

### 3.1 Inorganic Lead and Tin Halide Perovskites

Among the most extensively studied perovskite systems for solar energy conversion, inorganic lead halide perovskites such as CsPbBr_3_ and CsPbI_3_ have attracted widespread attention owing to their excellent optoelectronic properties. Experimentally, their phase transitions are closely associated with the tilting of PbX_6_ octahedra, governed by soft phonon modes that typically become overdamped near structural instabilities. To quantitatively resolve the dynamic local octahedral tilting in CsMX_3_ (M = Sn, Pb; X = Cl, Br, I), Wiktor et al. employed NEP to perform large-scale MD simulations over 20–620 K and up to 100 ns and compared the impact of different exchange–correlation functionals used to construct the training sets.^80^ They showed that SCAN + rVV10 and vdW-DF-cx yield transition temperatures and tilting angle distributions in agreement with the experiment, whereas PBE and PBE + D3 exhibit substantial deviations. A clear chemical trend emerged: larger halogens and Pb (vs Sn) favor stronger octahedral tilting and higher transition temperatures. The discrepancy between average and simultaneous structures was traced to persistent short-range order in the high-temperature phase, with correlation lengths on the order of ∼20 Å.

The accurate and long-time-scale simulations enabled by MLP also provide a powerful route to revisit conventional pictures of vibrational dynamics. In the standard view, phonons are considered well-defined quasiparticles characterized by distinct frequencies and lifetimes. Fransson et al. demonstrated a breakdown of this quasiparticle picture in CsPbBr_3_ by constructing MLPs and performing large-scale MD simulations in GPUMD.^81^ Their analysis focused on soft phonon modes associated with [PbBr_6_]^4–^ octahedral tilting during the cubic → tetragonal → orthorhombic phase transitions. Near the phase boundaries, these soft modes were found to be strongly overdamped and to remain overdamped up to 200 K above the cubic-to-tetragonal transition temperature. Moreover, the relaxation time was observed to increase with damping, in contrast to the conventional expectation. The two transitions also display distinct characteristics: the cubic-to-tetragonal transition is close to first-order, while the tetragonal-to-orthorhombic transition is more continuous. Crucially, these simulations show that the high-symmetry cubic phase of CsPbBr_3_ is stabilized by the vibrational entropy associated with soft, strongly anharmonic modes rather than by enthalpy, given that the cubic structure is often energetically unstable at 0 K. This result highlights that MLP-driven MD can preserve key thermodynamic relationships: by capturing the full anharmonicity of the potential energy surface, such trajectories naturally reproduce the entropy-driven stabilization, which is inherently inaccessible to static calculations or harmonic approximations.

Traditionally, phase behavior in perovskites has often been discussed from a global symmetry perspective, assuming a disordered distribution of octahedral tilting angle. However, recent experiments have uncovered complex soft phonon modes and planar correlation patterns in MAPbI_3_ and CsPbBr_3_, pointing to locally asymmetric structures even within high-temperature phases. In CsPbI_3_, such local asymmetric dynamics hidden in a high symmetrical global vision have been recognized with MLPs. For example, near the δ-α phase transition of CsPbI_3_, MD simulations based on NEP trained on the vdW-DF-cx database were combined with inelastic neutron scattering to validate the predicted vibrational behavior.^83^ The room-temperature-stable δ-phase was found to exhibit relatively weak anharmonicity and phonon modes with weak temperature dependency, while the high-temperature α-phase shows strongly anharmonic heavily damped modes. Interestingly, even at ∼150 K above the δ-to-α transition temperature, the [PbBr_6_]^4–^ octahedral tilting modes in the α-phase remain overdamped, with relaxation times of 2–4 ps, indicating persistent structural fluctuations. This work provides evidence for a flat energy landscape and locally noncubic structure in α-phase CsPbI_3_, emphasizing the distinction between spatially averaged crystallographic symmetry and the rich, heterogeneous local environment captured by MLP-based simulations.

On the other hand, Baldwin et al. investigated and visualized the intricate temporal and spatial correlations in the local structure of CsPbI_3_.^82^ They developed MLP based on the atomic cluster expansion (ACE) framework and focused on the cubic, tetragonal, and orthorhombic phases. Their results show that the octahedral tilting angles exhibit strong correlations in both temporal and spatial domains. In the time domain, the tilts display a superposition of high-frequency vibrational motion and low-frequency hopping in the cubic phase. In the spatial domain, the tilting angles form 2D planar correlation patterns: for each tilt direction, correlation persists over long ranges along the direction perpendicular to the tilt plane but decays along the tilt direction itself (Figure 3). Furthermore, low-symmetry structures characteristic of tetrahedral and orthorhombic phases are found to transiently emerge within structures nominally classified as cubic or tetrahedral. The high accuracy of the ACE-MLP, combined with large-scale simulations involving over 69,000 atoms for trajectories up to 1 ns, enables subpicosecond resolution of both vibrational and hopping dynamics, thereby revealing rich instantaneous local structures that are invisible in conventional temporal and spatial averages.

**Figure 3:**
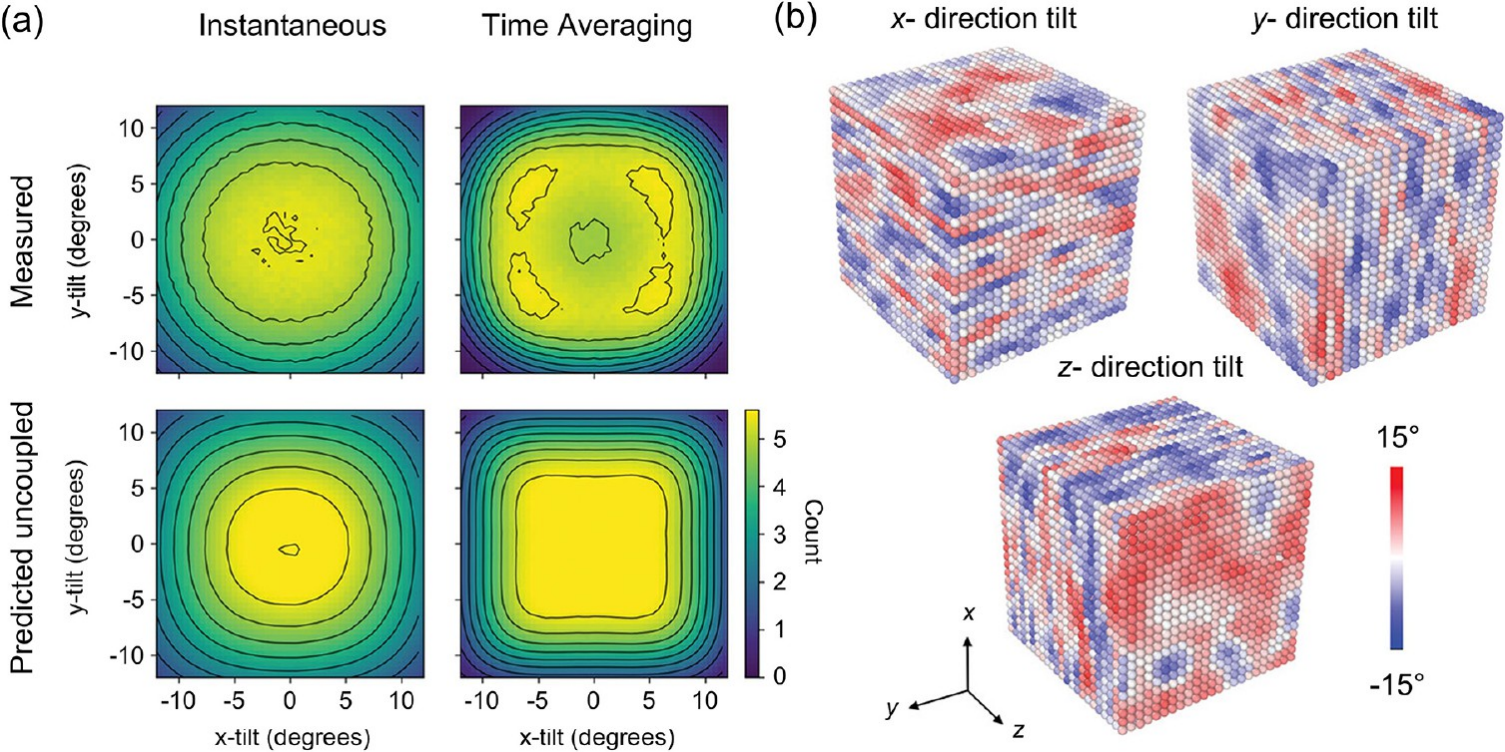
(a) Heat maps of tilting angles of [PbI_6_]^4–^ octahedra along the x-axis and *y*-axis (θ_*x*_ and θ_*y*_) in the cubic phase at 550 K for CsPbI_3_. (b) Visualization of the time-averaged tilting structure for CsPbI_3_. Adapted with permission from ref 82. Available under the terms of the Creative Commons CC BY license. Copyright 2024 Baldwin et al.

From the perspective of constructing an effective, transferable, and reliable model for accurately predicting phase properties, recent work has focused on the design of training sets for MLPs. Braeckevelt et al. showed that, despite fluctuations in local geometric structures across different phases, their MLPs can still reliably predict the phase transition temperature of CsPbI_3_, yielding a γ → δ transition range of 548–599 K that is in very good agreement with the experiments.^84^ The fidelity of the MLPs is illustrated in Figure 4, which compares results obtained with different training set sizes against DFT calculations using CP2K as a benchmark. Larger supercells generally show better agreement with high-level reference calculations. In addition, a group of commonly used DFT functionals were compared in terms of their predicted total free energy differences. While CP2K and the MLPs show only minor deviations from each other, the choice of functional leads to significant variation, resulting in biases of 100 K in the transition temperature. Random phase approximation (RPA) with Hartree–Fock (HF) energy calculations was therefore used as a reference. This study highlights the reliability of MLPs in solving numerical regression problems, which is exactly the strength of deep neural network frameworks such as SchNet. Although the inclusion of three distinct phases supports good transferability, the need for high-quality training data remains central and is still an active topic of discussion.

**Figure 4:**
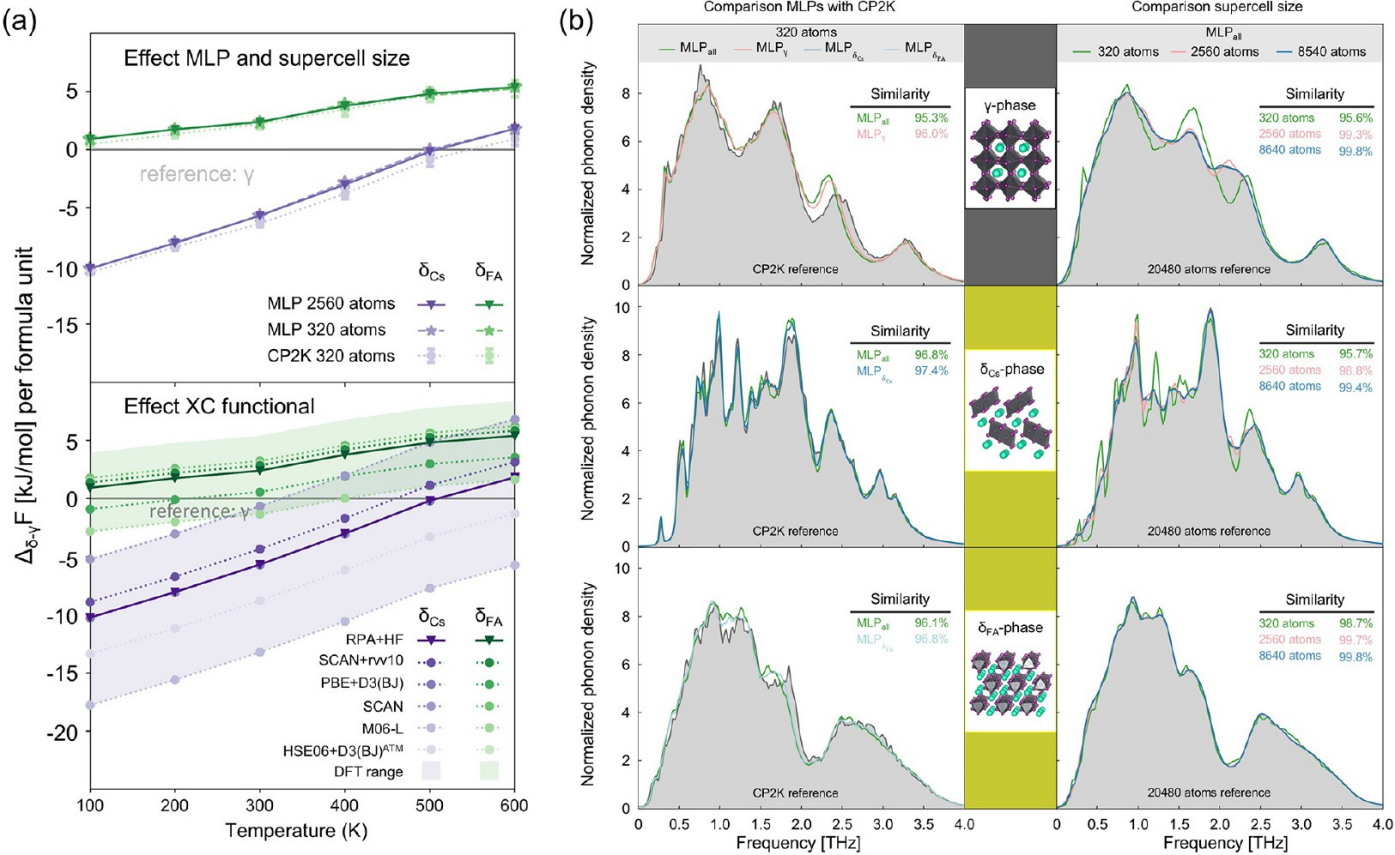
(a) Total free energy difference of the δ_Cs_ and δ_FA_ phases relative to the γ phase as a function of temperature, highlighting the effects of the MLP, supercell size, and exchange–correlation (XC) functional. (b) Vibrational density of states (VDOS) for different phases, comparing results obtained with different MLPs to those from CP2K. Adapted with permission from ref 84. Copyright 2022 American Chemical Society.

Another practical question in applying the MLPs concerns the optimized hyperparameters to simultaneously achieve consistency with both ab initio calculations and experimental observations. Fransson et al. systematically analyzed common parameters such as heating-cooling rate, finite-size effects, model uncertainty, and the influence of different exchange-correlation functionals.^85^ They constructed NEP trained on data sets generated with various functionals and performed MD simulations using GPUMD on a system containing over 60,000 atoms trajectories spanning hundreds of nanoseconds. Their results suggest that reliable transition temperatures require simulations with more than ten thousand atoms and a heating-cooling rate less than 60 K/ns, with an intrinsic model uncertainty of around ±30 K. All models trained on different functional data sets qualitatively reproduce the orthorhombic → tetragonal → cubic transition sequence but systematically underestimate the absolute transition temperatures. Among the functionals examined, vdW-DF-cx and SCAN + rVV10 give the best agreement with experiment. This work provides a quantitative assessment of functional-dependent uncertainties in perovskite and offers guidance for optimizing model choices to balance accuracy and computational cost.

To quantitatively characterize perovskite lattice dynamics, Liang et al. developed PDynA, a package of descriptors designed for automatic postprocessing of MLP-based MD trajectories.^86^ The descriptors are tailored to capture dynamic local structures in perovskites. They validated PDynA using Gaussian approximation potential (GAP)-based MLPs for MAPbBr_3_ and atomic cluster expansion (ACE)-based MLPs for CsPbI_3_ and successfully identified key local phenomena. For MAPbBr_3_, the force field reproduces the experimentally known α → β transition temperature of ∼225 K, while it underestimates the β → γ transition (∼110 K). For CsPbI_3_, [PbI_6_]^4–^ octahedral tilting was observed, leading to locally asymmetric regions near phase transition, which could be quantitatively analyzed using the octahedral-tilting descriptors.

In addition to lead-based compounds, lead-free perovskites such as CsSnBr_3_ and CsSnI_3_ have been explored as environmentally friendly alternatives, with their phase stability examined via P–T phase diagrams and their thermoelectric properties evaluated using the NEP framework.^87^ Although the calculated transition temperatures are generally underestimated compared to the experiments, an important conclusion is that at elevated pressures the γ-phase becomes more stable due to the disappearance of soft modes, thereby enhancing structural stability. For *n*-doped CsSnI_3_, the calculated *ZT* value of 0.184 at 3 GPa and 400 K falls within the experimentally reported range of 0.08–0.21. The large-scale MD simulations with 16,000 atoms based on NEP also further elucidate the structural evolution under different pressures, revealing microscopic mechanisms behind soft-mode suppression and the associated stabilization of the perovskite phase.

Building on these comparative studies, we provide a critical assessment of the choice of DFT functionals for constructing MLPs aimed at predicting phase behaviors in inorganic perovskites. Standard GGA functionals combined with dispersion corrections (e.g., PBE + D3) often systematically underestimate the phase-transition temperatures. This deficiency is commonly attributed to an inadequate description of dispersion, which can bias equilibrium lattice volumes and soften the effective stiffness of octahedral tilting modes that govern the displacive transitions. By contrast, SCAN + rVV10 demonstrates markedly improved agreement with experimental lattice parameters and transition temperatures. Although higher-level approaches such as the random phase approximation (RPA) can provide superior energetic benchmarks, they remain computationally prohibitive for generating the tens of thousands of configurations required to train robust MLPs. Therefore, for practical applications focusing on phase stability and thermodynamics, we identify SCAN + rVV10 as the most compelling current choice, offering the best balance between thermodynamic accuracy and the computational feasibility of large-scale data generation.

### 3.2 Hybrid Lead Halide Perovskites

Hybrid lead halide perovskites, in which the A-site is occupied by organic cations such as MA^+^ or FA^+^, also exhibit excellent optoelectronic properties, tunable bandgaps, and outstanding energy conversion efficiency for photovoltaic applications. However, their practical deployment is still limited by issues of phase stability and carrier lifetime, which ultimately determine long-term device reliability. To address these challenges, researchers have extensively investigated both macroscopic phase diagrams and microscopic lattice and carrier dynamics, as well as low-temperature phase geometry.

Constructing global phase diagrams in a high-dimensional parameter space is essential for rational device design but is computationally intensive. The NEP potentials combined with umbrella sampling have enabled large-scale simulations to map the free-energy landscape of CsPbBr_3_ and MAPbI_3_ in Glazer space, thereby revealing their phase transition pathways and metastable behaviors.^89,90^ For CsPbBr_3_, the orthorhombic-to-tetragonal phase transition is found to be continuous, whereas the tetragonal-to-cubic transition displays first-order character with a small latent heat. The corresponding free-energy surface is extremely flat, making phase transitions readily accessible. In contrast, MAPbI_3_ exhibits a more complex free-energy landscape, leading to an extended metastable region at low temperature where multiple competing polymorphs can coexist. This enables the construction of a metastable phase diagram for MAPbI_3_, providing insight into the structural stability of both inorganic and hybrid perovskites.

Motivated by the search for optimal compositions for stable perovskite solar cells, Luo et al. extended this perspective to a high-dimensional compositional space by analyzing the Gibbs free energy of the FA_*x*_Cs_1–*x*_Pb­(I_*y*_Br_1–*y*_)_3_ system.^91^ They developed a neural network potential to address two key tasks: (i) searching composition space for the most stable structures and (ii) inverse designing the most stable composition that satisfies a target bandgap. The training set was generated from DFT calculations combined with stochastic surface walking (SSW) sampling, covering more than 6 × 10^5^ configurations. Their results indicate that Br-rich compositions tend to phase-segregate with negative Gibbs free energy, while I-rich and FA-rich compositions are more stable. Introducing Cs at the A-site at the levels below 40% mitigates Br-induced segregation, with 20% Cs identified as optimal. By combining stability and bandgap calculations, they proposed two promising compositions: FA_0.63_Cs_0.37_Pb­(I_0.88_Br_0.12_)_3_ and FA_0.63_Cs_0.37_Pb­(I_0.69_Br_0.31_)_3_. The former has a bandgap of 1.7 eV, is more stable, and is suitable for Si/perovskite tandem cells, and the latter has a bandgap of 1.8 eV, appropriate for perovskite/perovskite tandems, but is thermally less stable and requires kinetic stabilization.

Beyond global composition optimization, considerable attention has also been paid to how different A-site cations and mixed halide distributions affect transition pathways and local structures. To capture these effects, Liang et al. constructed the MLP based on MACE architecture and applied it to three prototypical families (CsPbX_3_, MAPbX_3_, and FAPbX_3_, with X = I and Br) for mapping their phase diagrams (Figure 5).^88^ MD simulations show that the A-site cations strongly dictate the phase transition pathways. Specifically, MA^+^ tends to hinder the β → γ transition, while FA^+^ favors the formation of cubic phase at low temperature. At the same time, the halide environment and its local dynamics are shown to modulate the macroscopic phase behavior. Uniform halide mixing leads to I-rich octahedra that preferentially tilt toward γ-like configurations, whereas Br-rich regions bias the octahedra toward β-like tilts. Halide segregation further induces anomalous tilting modes, underscoring the intricate coupling between local composition, lattice dynamics, and emergent phase behavior in hybrid perovskites.

**Figure 5:**
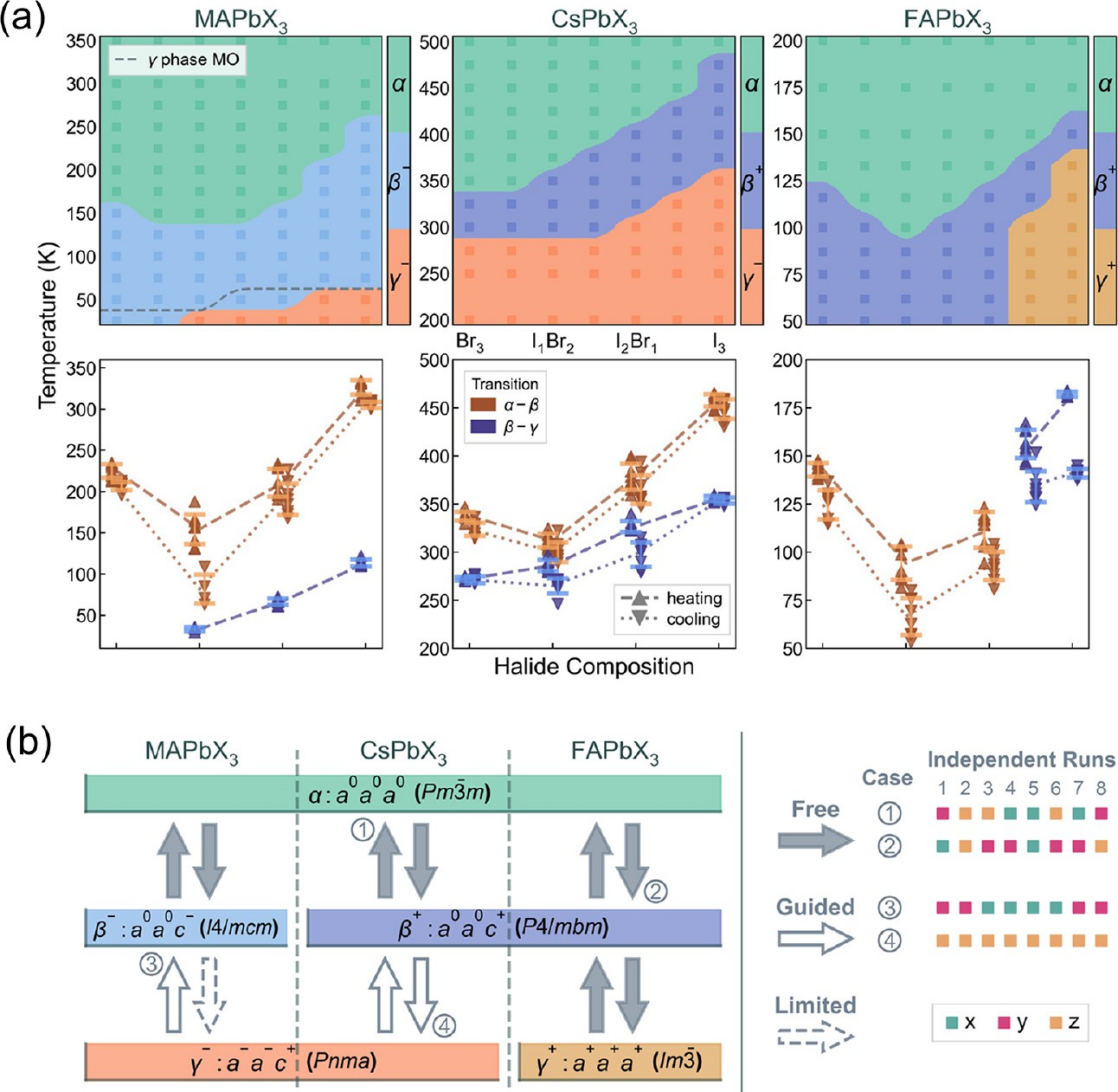
(a) Equilibrium-based phase diagrams and transition temperatures extracted from heating and cooling and (b) the main transition pathways for CsPbX_3_, MAPbX_3_, and FAPbX_3_. Adapted with permission from ref 88. Copyright 2025 American Chemical Society.

The validated MLPs reveal previously unresolved aspects of phase stability in halide perovskites, including accurate transition temperatures and a clear picture of how local tilt dynamics evolve across a morphotropic phase boundary. Recently, a morphotropic phase boundary (MPB) in the MA_1–*x*_FA_*x*_PbI_3_ was identified at ∼27% FA composition by Hainer et al.^92^ Using NEP trained on SCAN + rVV10 data, they constructed a phase diagram in Glazer space (Figure 6), showing that MPB separates the *a* ^0^ *a* ^0^ *c* ^–^ tilting mode of MAPbI_3_ from the *a* ^0^ *a* ^0^ *c* ^+^ tilting mode of FAPbI_3_. Near the boundary, the free energies associated with the M mode and R tilt modes are nearly degenerated, leading to alternating layered structures accompanied by strong overdamping. Electronic properties are also markedly affected: the fluctuation of valence band maximum (VBM) peaks close to MPB, reflecting enhanced electron–phonon coupling. The existence of MPB thus provides a powerful handle for compositional tuning of both stability and optoelectronic properties.

**Figure 6:**
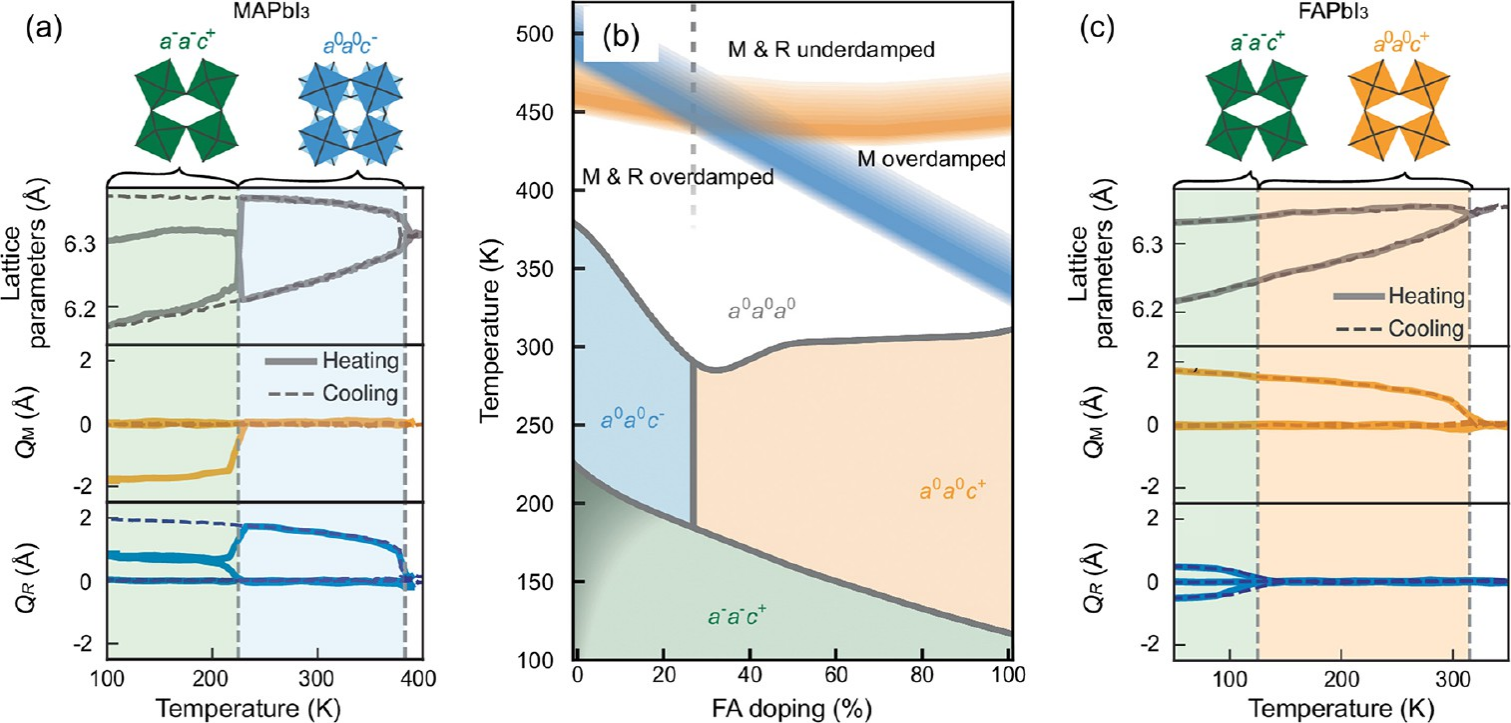
(a) Lattice parameters and activation of the M and R phonon modes during heating and cooling of MAPbI_3_. (b) Predicted phase diagram of MAPbI_3_, labeled in Glazer notation, constructed from the activation behavior of these phonon modes. (c) Lattice parameters and activation of M and R phonon modes during heating and cooling of FAPbI_3_. Adapted with permission from ref 92. Available under a Creative Commons Attribution 4.0 International License. Copyright 2025 Hainer et al.

From a macroscopic perspective, the phase diagram encodes stable structures and transition pathways as functions of composition and temperature. However, a deeper understanding requires resolving low-temperature phases of hybrid perovskites, where the coupling between octahedral tilts and the orientational freezing of organic cations is essential to explain the dynamic disorder and guide strategies for device stabilization. Using deep potential molecular dynamics (DPMD) and isothermal–isobaric NPT simulations based on MLP, it was known that MAPbI_3_ and FAPbI_3_ spontaneously form hybrid nanodomains at low-temperature γ-phase.^93^ Within a single [PbI_6_]^4–^ octahedral rotation domain, multiple nanodomains of molecular rotations coexist. The underlying mechanism involves two types of coupling: between neighboring inorganic cages and between each organic molecule and its host cage. The organic–inorganic coupling is mediated by hydrogen bonding (HB), and because MA forms stronger HBs than FA, the characteristic molecular-rotation domain size is smaller in MAPbI_3_ (2.5 nm) than in FAPbI_3_ (5 nm). The MLP-based simulations not only clarify this link between the coupling mechanism and the HB energetics but also connect the rotation barrier of organic cations to domain size (Figure 7), thereby providing microscopic insight into the nanodomain phase behavior observed experimentally in FAPbI_3_.

**Figure 7:**
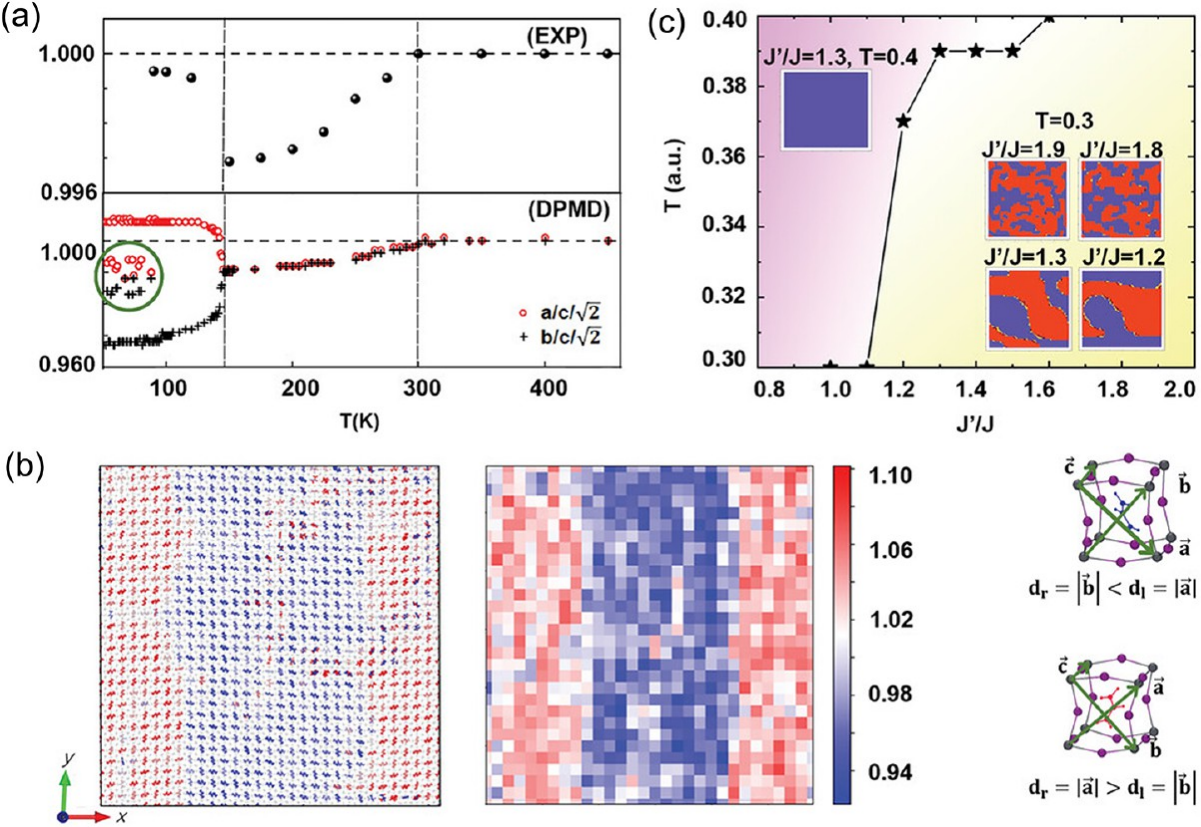
(a) Tetragonal lattice parameter ratio from synchrotron XRD experiments and simulated by DPMD. (b) Schematic illustration of the supercell with multidomains. (c) The standard Monte Carlo simulation results of the coupled-lattice Ising model at different *J* ^*′*^/*J* and temperatures. Adapted with permission from ref 93. Copyright 2023 John Wiley & Sons, Inc.

A further critical role of MLPs is their ability to resolve long-standing structural and dynamic controversies in low-temperature phases through sufficiently long and large-scale simulations. Dutta et al. recently addressed such controversy on the γ-phase FAPbI_3_ using the fourth generation of NEP.^90^ Their results indicate that the true ground state of FAPbI_3_ is an *a* ^–^ *b* ^–^ *b* ^–^ tilt pattern with antiferroelectric character, whereas during cooling, the system can become trapped in a higher-energy *a* ^–^ *a* ^–^ *c* ^+^ state. This trapping is associated with the freezing of FA orientations, which prevents the system from surmounting the free-energy barrier and reaching the ground state. This provides an explanation for long-standing discrepancies among experimental reports and highlights the importance of metastable frozen configurations in governing the low-temperature phase behavior of FAPbI_3_.

One of the distinctive features of hybrid perovskites is the presence of HBs between organic cations (with the −NH_3_ or −NH_2_ group) and PbX_6_ octahedra. These HBs modulate the local orientation dynamics of cations and thereby contribute to overall phase stability.^94^ Garrote–Márquez et al. investigated this mechanism using MLP-based simulations of MAPbBr_3_. Focusing on the N–H···Br and C–H···Br interactions across 70–350 K and spanning the orthorhombic, tetragonal, and cubic phases, they found that HB lifetime decreases with increasing temperature, from ∼7.6 ps at 70 K to ∼0.16 ps at 350 K. The HB breaking kinetics follow Arrhenius behavior with activation energies of 0.02–0.03 eV for all the phases. Furthermore, N–H···Br bonds are more stable than C–H···Br, exhibiting longer lifetimes. The picosecond HB dynamics were shown to have significant influence on vibrational modes, as evidenced by power spectra derived from the trajectories, underscoring the intimate coupling between local HBs, lattice dynamics, and phase stability in hybrid perovskites.

### 3.3 Two-Dimensional Layered Hybrid Perovskites

2D hybrid perovskites, with their layered structures separated by organic spacers, have attracted increasing attention. Compared with their less compositionally flexible 3D counterparts, these quasi-2D systems offer enhanced stability and highly tunable optoelectronic properties through precise control over both inorganic thickness and organic spacer layers. MLP-driven MD simulations have been used to elucidate how organic–inorganic interactions govern dynamic distortions and the thermal properties in quasi-2D Ruddlesden–Popper perovskites, A_2_PbI_4_ (A = BA, PEA), in comparison with 3D MAPbI_3_.^95^ For BA_2_PbI_4_, a phase transition occurs at 274 K, driven by the disordering of BA and straightening of the Pb–I framework, allowing deeper penetration of the ammonium group into the inorganic cage at high temperature. The two Ruddlesden–Popper phases exhibit lower thermal conductivity than MAPbI_3_; although BA_2_PbI_4_ shows stronger structural distortions, PEA_2_PbI_4_ has the lowest thermal conductivity. PEA_2_PbI_4_ and BA_2_PbI_4_ also possess higher heat capacities than MAPbI_3_, but with distinct temperature dependencies: the heat capacity of PEA_2_PbI_4_ increases monotonically with temperature, while that of BA_2_PbI_4_ tends to be flat above 373 K. In addition, the density of state at the valence band is strongly influenced by distortions of the inorganic layer, which are themselves indirectly driven by the dynamics of the organic spacers. These distortions, occurring on hundreds of picosecond time scales, are accurately captured by MLP-based simulations, indicating how the organic–inorganic coupling drives the structural fluctuations, thermal transport, and electronic states.

Understanding this so-called templating effect is important: experiments have shown that the organic spacer layers are not simply passive structural supports but also actively modulate octahedral tilting and phase transition in the inorganic layers. A recent study examined how different organic spacers (PEA, PMA, BA, and MA) and the number of perovskite layers (*n*) influence phase transition, octahedral tilting modes, and ultimately the electronic properties of 2D perovskites.^96^ Using NEP, they showed that surface layers exhibit significantly higher transition temperatures than the interior; for example, in PEA/MAPbI_3_, the surface transitions around 450–470 K, while the interior transitions near 400 K. As *n* increases up to 30, the transition temperature converges to 370 K, matching that of bulk MAPbI_3_. Comparing different spacers, PEA and PMA raise the transition temperature above the bulk value, BA nearly induces almost no difference, while MA lowers it relative to bulk MAPbI_3_ (Figure 8). These findings establish a clear templating strategy: by choice of organic spacer and layer thickness, one can deliberately engineer the structural dynamics and thus the optoelectronic properties of 2D hybrid perovskites.

**Figure 8:**
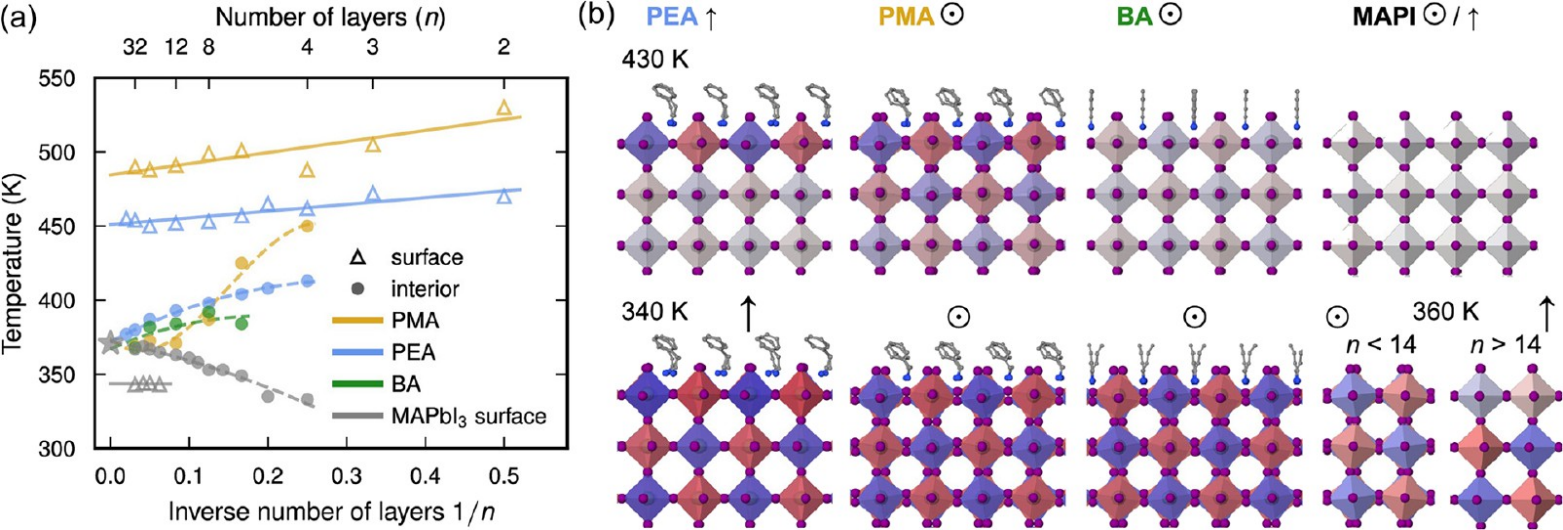
(a) Transition temperatures as a function of the number of layers and (b) average atomic configurations at 430 K and 340/360 K for PEA, PMA, and BA-based 2D hybrid perovskites, as well as MAPbI_3_ surfaces. Adapted with permission from ref 96. Copyright 2024 American Chemical Society.

Surveying recent progress across perovskite materials, we observe systematic differences in the capability of current MLPs to capture phase transitions. For inorganic and hybrid 3D perovskites, MLPs have achieved high fidelity in reproducing polymorphic stability and the observed sequence of phase transitions. This success largely reflects the displacive nature of these transitions, which are driven by octahedral tilting and lattice distortions rather than bond breaking. Accordingly, modern active-learning strategies (e.g., DP-GEN) are well suited for efficiently sampling the largely continuous configuration spaces. In contrast, accurately describing reconstructive transitions, including degradation from the photoactive black phase to the nonperovskite yellow phase, remains a grand challenge. These processes involve bond breaking, large structural rearrangements, and high activation barriers, yielding rare-event pathways that are difficult to sample extensively. For 2D perovskites, MLP applications to phase stability are considerably less mature. A central bottleneck is the vast chemical and configurational space introduced by the organic spacers. Unlike the relatively simple A-site cations in 3D systems, the organic spacers exhibit substantial structural diversity and rich conformational flexibility, which dramatically expands the configurational boundaries. As a result, achieving high transferability for polymorphic stability in 2D perovskites will likely require significantly larger and more heterogeneous data sets, alongside the development of universal foundation models that can generalize across diverse organic functionalities.

## 4 Ion Migration and Point Defects

### 4.1 Ion Migration

Ion migration in halide perovskites refers to the transport of halide ions or halide-site defects (e.g., iodide vacancies) away from their equilibrium positions under external stimuli. Such conditions include high defect concentrations, thermal fluctuations, and external electric fields, which are common phenomena during the intrinsic device operation. Ion migration can intrinsically degrade the lattice, introduce complex interactions among mobile ions or defects, generate local band bending, and thereby alter the dynamic electronic response. MLP-based simulations have been increasingly used to disentangle the various factors that govern this migration behavior.

In bulk γ-CsPbI_3_, Arber et al. examined how B-site dopants influence iodine vacancy migration.^97^ They considered Ba^2+^, Cu^2+^, Zn^2+^, Ge^2+^, Cd^2+^, and Sn^2+^ at low concentration (3%) and employed large-scale MD simulations driven by MLP with a MACE framework, covering temperatures from 300 to 500 K over 80 ns. From these trajectories, iodide diffusion coefficients and activation energies were extracted (Figure 9a), and representative iodide migration paths were visualized (Figure 9b). Somewhat unexpectedly, most dopants had only a minor impact on the migration barriers: Sn^2+^ slightly increased the barrier, whereas Zn^2+^ even decreased it. The iodine vacancies were confirmed to migrate along the edge of [PbI_6_]^4–^ octahedra. This work provides a microscopic, time-resolved view of iodide migration in doped γ-CsPbI_3_ under near-room-temperature conditions, illustrating how subtle the dopant effect can be.

**Figure 9:**
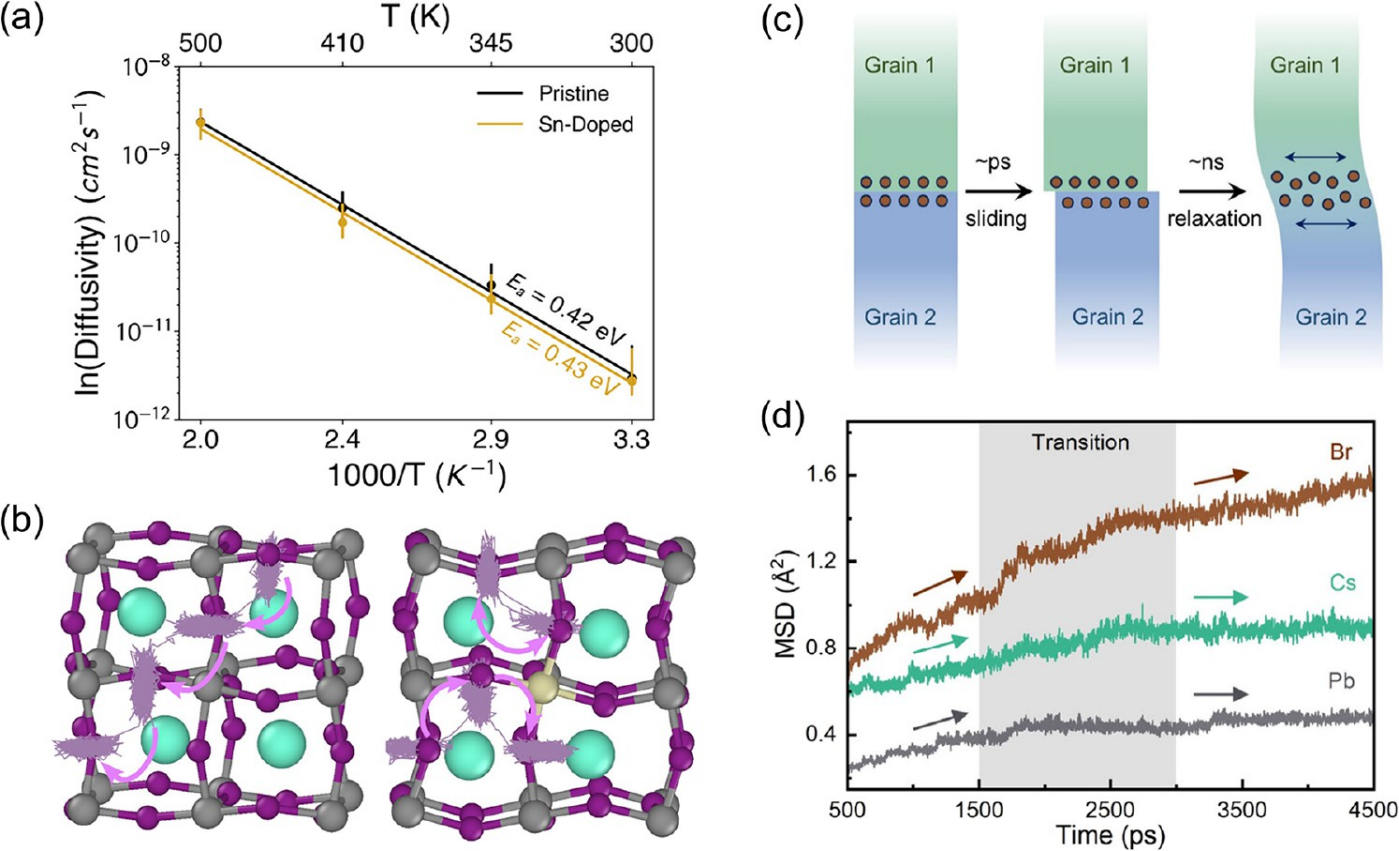
(a) Arrhenius plots of the calculated diffusion coefficients for pristine and 3% Sn-doped γ-CsPbI_3_. (b) Iodide-ion trajectories from the MD simulations superimposed on the relaxed γ-CsPbI_3_ lattice. Adapted with permission from ref 97. Copyright 2025, American Chemical Society. (c) Scheme of the structural evolution of the GB model. (d) Mean squared displacement (MSD) of different elements as a function of time, referenced to the structure at 500 ps. Adapted with permission from ref 98. Copyright 2024 American Chemical Society.

The 3D migration channels for bromide ions along a Σ5(120) GB of CsPbBr_3_ have also been recently elucidated using MLPs.^98^ In this study, a DP model trained on AIMD/DFT data was used to perform large-scale MD simulations involving 6400 atoms over 10 ns at 300, 350, and 400 K. The GB structure itself evolves with temperature and time and continuously reshapes the available migration pathways. By analyzing dynamical descriptors such as the mean squared displacement (MSD), radial distribution function (RDF), and Br coordination numbers, the spatial distribution of migratable Br ions was mapped (Figures 9c,d). The Br ion preferentially migrates toward the GB core and along the boundary plane. While both bulk and GB diffusion contribute to 3D transport, the GB remains the dominant fast-migration channel. Increasing temperature activates more Br^–^ ions and enhances the overall migration rate, which follows Arrhenius behavior and confirms that activation energy at the GB is much lower than in the bulk. Local structural analysis further reveals that GB regions enriched in Pb and depleted in Cs favor Br migration, as Br can coordinate with more Pb and fewer Cs ions in these environments. These MLP-based insights point to practical strategies for suppressing ion migration, such as tuning GB stoichiometry, to enhance the operational stability of halide perovskite devices.

Tyagi et al. investigated defect migration in the bulk CsPbI_3_ system as a function of defect charge state.^99^ Iodide vacancy (V_I_) and iodide interstitial (I_i_) in three charged states (neutral, negatively charged, and positively charged) were examined using MLP based on Gaussian approximation potentials (GAPs), which was then employed in MD simulation on 2 ns from 500 to 600 K. The key observations (e.g., diffusion coefficients and activation energies) were used to rank the migration ability of each defect species (Figure 10). The charge state was found to have a pronounced effect on the migration rate: neutral interstitial (I_i_^0^) diffuses the fastest, with neutral vacancy (V_I_^0^) being roughly an order of magnitude slower; I_i_^–^ and V_I_^+^ exhibit similar diffusion coefficients. These results provide a new vision of the experimentally observed decrease in ionic conductivity when moving from an iodine-poor to an iodine-rich condition, which is attributed to changes in the dominant defect charge states. Methodologically, the MLP stands out because separate potentials can be constructed for different charge states via multiple trainings, enabling realistic modeling of device operation under nonequilibrium conditions.

**Figure 10:**
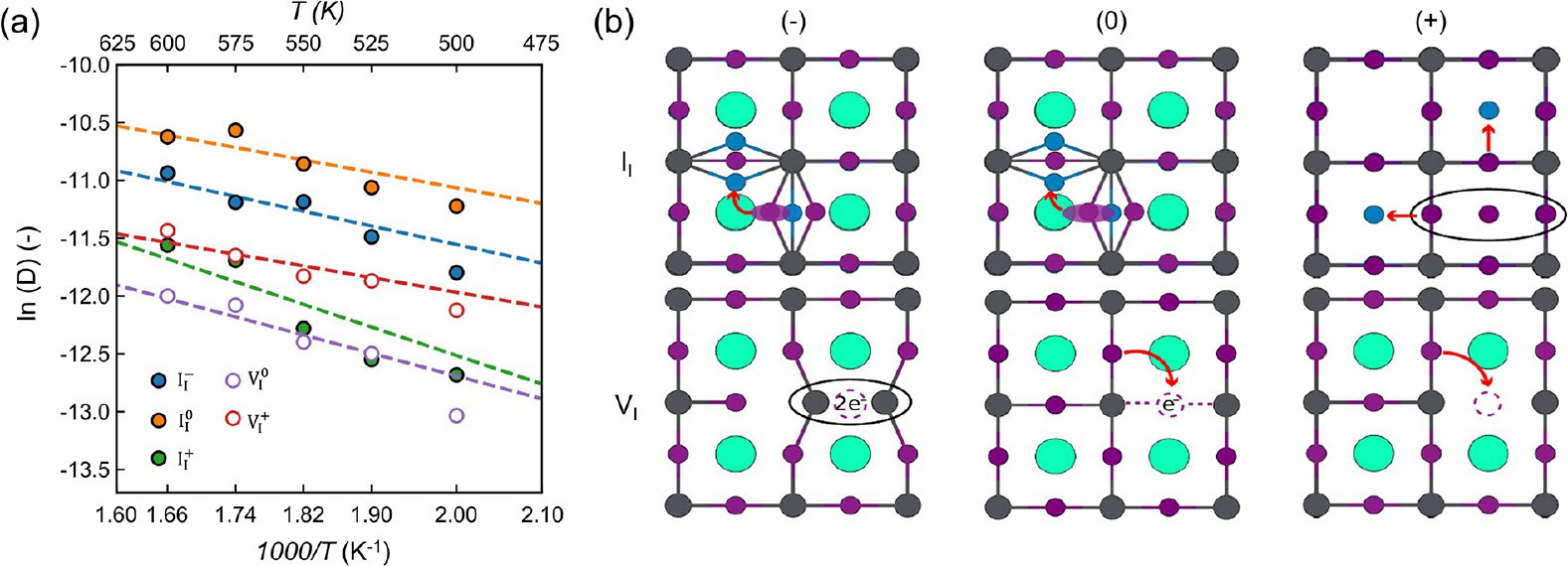
(a) Temperature-dependent diffusion coefficients of halide point defects obtained from MLP-based MD simulations. (b) Schematic representations of the diffusion pathways of iodide interstitials and vacancies in their different charge states. Adapted with permission from ref 99. Copyright 2025 American Chemical Society.

The impact of ion migration on local structure and electronic properties has also been explored in detail. Hao et al. reported that interstitial iodine in bulk MAPbI_3_ exerts a synergetic influence on structural distortions and carrier recombination.^100^ They identified a three-stage migration pathway for I_i_, in which iodine trimers form during the initial and final stages, causing strong local distortions. Along this pathway, defect-induced trap states evolve dynamically: a deep trap state appears within the band gap when I_i_ occupies the vertical (V) and parallel (P) configurations, whereas at the geometric middle point of the path, the traps become shallower and shift below the VBM, giving rise to deep-shallow-deep stages. In contrast to the common belief that iodine migration is uniformly detrimental to the carrier lifetimes, their calculations show that the shortest pure dephasing time (16 fs) and longest lifetime of charge carriers (∼8 ns) appear at the middle stage, indicating that I_i_ can be transiently benign and may even delay nonradioactive recombination. All these insights are derived from high-accuracy DP models used in DPMD simulations at a time scale of 5 ns, providing a microscopic picture of the coupled structure-electronic-dynamical landscape in hybrid perovskites.

### 4.2 Point Defects

Ionic point defects have been frequently discussed both computationally and experimentally to criticize the thermal stability of perovskites. The MLPs provide a systematic route to quantify how defect concentration and defect locations affect phase behavior in CsPbI_3_.^101^ It is shown that multiple DFT-based training sets and multiscale MD simulations were used to determine the minimal system size and simulation time required for reliable predictions; it was concluded that at least ∼3600 atoms and ∼0.25 ns are necessary to achieve converged results. A DP model trained within the DP-GEN active-learning framework, using 7730 structures covering multiple phases and temperatures, was shown to outperform both Lennard-Jones (LJ) models and direct AIMD in terms of accuracy and transferability.

The calculated shift temperature for CsPbI_3_ was 1160 K, higher than the 850 K reported by experiments. This discrepancy was attributed to the breaking of Pb–I bonds and gradual loss of long-range order at the theoretical transition temperature. Among the three types of defects (V_Cs_, V_Pb_, and V_I_), the V_Cs_ have the most detrimental effect on stability, with the lowest defect tolerance concentration of 0.32%, while the lattice is comparatively tolerant to the V_I_ in the bulk. Surface defects were also examined and found to exacerbate instability relative to bulk defects. Specifically, surface V_Cs_ becomes critical at concentrations below 15%, while V_Pb_ dominates the destabilization when its surface concentration is higher than 20%. Overall, the study concludes that the surface defect causes more severe instability than bulk defects. This work also showed that the MD simulations, limited to ∼1 ns, may still miss slower breakdown and reconstruction processes, underscoring the need for even longer MLP-based trajectories to fully capture long-time-scale defect-driven degradation.

Defect fluctuations also exert significant influence on the trap states in CsPbI_3_, as demonstrated using the MLPs based on neural networks coupled to three different MD methods.^102^ The MLP-MD was employed to access long-time-scale defect-induced structural fluctuations; AIMD was used to validate representative configurations with high accuracy along crucial trajectories, and nonadiabatic molecular dynamics (NAMD) combined with TD-DFT enabled the charge dynamics analysis, including nonradiative trapping and recombination, nonadiabatic coupling, and pure dephasing times. Figure 11 illustrates the structural evolution during 10 fs of the MLMD window. For the Cs_I_ defect, a stable I–I–I trimer forms, generating deep trap states and enhancing nonadiabatic coupling, thereby accelerating recombination and yielding a carrier lifetime of ∼937 ps. In the case of V_I_, a Pb–Pb dimer emerges; shallow trap states disappear when the Pb–Pb distance increases and recover as the dimer contracts. The shorter Pb–Pb distance, corresponding to stronger NAC, leads to a lifetime of 52 ps, while the weaker NAC interaction extends the lifetime to ∼855 ps. By contrast, I_i_ defects can survive for ∼800 ps, comparable to pristine CsPbI_3_. Figure 11 shows that I_i_ migrates extremely fast while its associated defect levels dynamically switch between CBM and VBM edges. Overall, this work demonstrates that electron–hole pair recombination rates are strongly governed by defect dynamics, which continuously modulate both the band gap and NAC. Complementing this dynamic picture, recent work employed equivariant MACE potentials to investigate chloride vacancies in CsPbCl_3_.^103^ Despite significant anharmonic fluctuations and the emergence of instantaneous deep-trap configurations, the thermodynamic charge-transition levels remain well-defined and consistent with equilibrium defect theory. This suggests that although transient dynamics are critical for carrier recombination, conventional static descriptors may still retain validity for predicting equilibrium defect energetics and concentrations.

**Figure 11:**
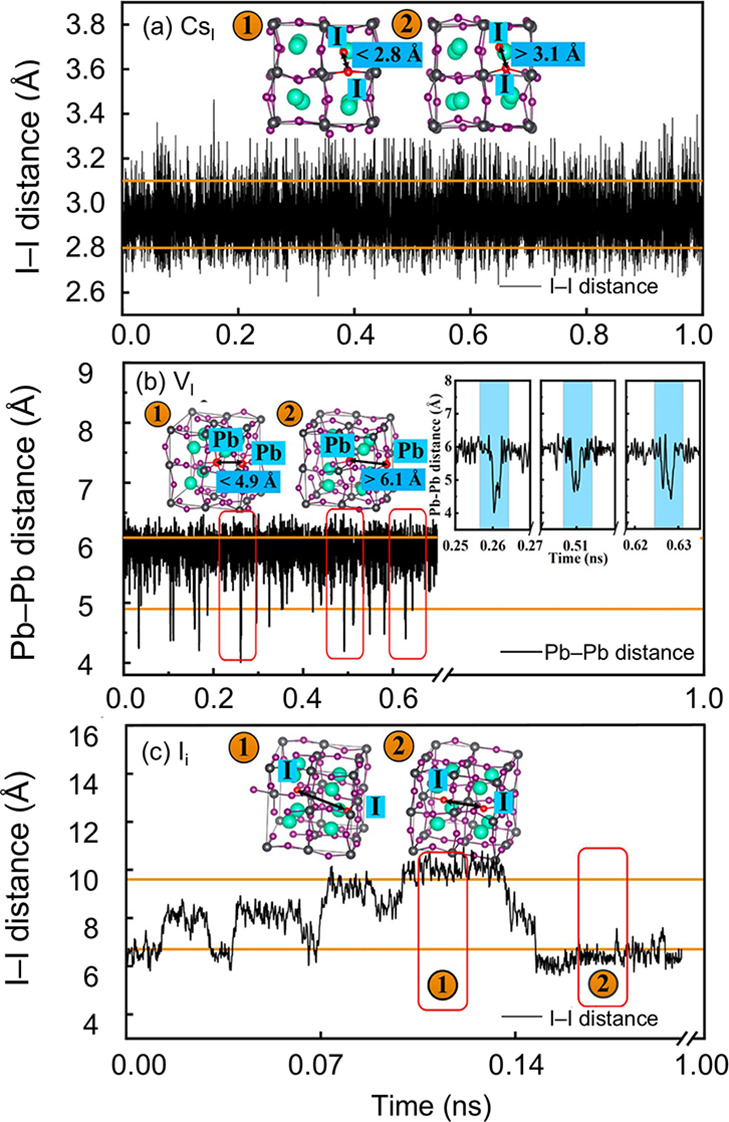
Evolution of the distance of (a) I–I nearby the defect in the Cs_I_ system, (b) Pb–Pb nearby the iodine vacancy in the V_I_ system, and (c) I–I around the interstitial iodine in the I_i_ system. Adapted with permission from ref 102. Copyright 2024 American Chemical Society.

The impact of defects on thermal transport has been investigated in CsPbBr_3_ by Han et al. using a DP model.^104^ The model was trained across four structural phases, using an active-learning strategy that systematically expanded training space to cover relevant regions of configuration space. Nonequilibrium molecular dynamics (NEMD) simulations of 5 ns revealed that the thermal conductivity of CsPbBr_3_ decreases with increasing V_Cs_ concentration. MSD distribution and VDOS for each ionic species were analyzed at 300 K. The results indicate that V_Cs_ defects induce significant Pb–Br cage distortions, which in turn enhance phonon scattering and shorten phonon lifetime, particularly at higher defect concentrations. In addition to DFT-level accuracy and computational efficiency, a notable advantage of this deep-learning potential is its ability to predict the energetics of V_Cs_ configurations that were not explicitly included in the training set, demonstrating impressive transferability.

The synergistic influence of GBs and point defects has been further investigated for Pb interstitials at a Σ5(210) GB of CsPbBr_3_.^105^ The study highlights three stages in the evolution of the local GB landscape. In the first 10 ps, the Pb–Pb–Pb trimer forms, opening the band gap. Over the subsequent 1 ns, the trap states oscillate between deep and shallow levels while the GB structure remains relatively stable. After 1.1 ns, sliding of GBs occurs, compressing Pb–Pb distances and stabilizing deep trap states that last for hundreds of picoseconds. The evolution of electronic structure was tracked along with the structural changes across 1.5 ns, enabled by DP-based MLPs trained on 40,000 DFT configurations spanning 100–1600 K, ensuring both accuracy and transferability. This work provides a compelling microscopic explanation for why carrier lifetimes and mobilities are strongly degraded at GB, as widely reported experimentally but previously lacking detailed atomistic simulation support.

## 5 Interfaces and Grain Boundaries

### 5.1 Interfaces

Surface interactions between perovskites and their ambient environments are a critical factor in determining the long-term stability of photovoltaic devices. Recently, we investigated the (100), (110), (111), and (210) facets of FAPbI_3_ in a moisture environment using an MLP-based method constructed by fine-tuning the DPA-2 pretrained model labeled with the SCAN-DFT database.^106^ We found that the PbI_2_-rich (100) facet is the most stable, exhibiting the lowest degradation rate and the highest activation energy, while the FAI-rich (110) facet tends to form HBs with water, which accelerates structural disorder and degradation (Figure 12). The (100) facet undergoes a distinct layer-by-layer degradation process. In addition, a newly introduced molecular orientation index (MOI) correlates FA surface orientational fluctuations with the degradation rate. The potential model enables efficient statistical analysis across a multiparameter space, including facet orientation, surface termination, temperature, and water densities, thereby linking macroscopic degradation behavior to microscopic dynamics and underscoring its value for designing atomistic surface passivation strategies.

**Figure 12:**
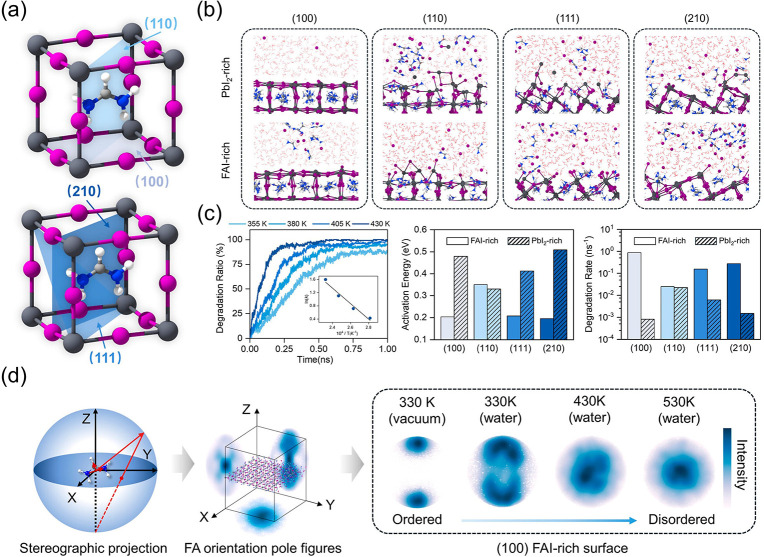
(a) Schematic diagram of the (100), (110), (111), and (210) facets. (b) Representative MD snapshots showing the degradation of these facets with different surface terminations under moisture at 430 K after 1 ns. (c) Time evolution of the degradation ratio of the outermost layer atoms on the FAI-rich (100) surface. (d) Schematic workflow constructing FA orientation pole figures, including identification of the FA orientation normal and stereographic projection of normal onto three planes. Adapted with permission from ref 106. Copyright 2025 American Chemical Society.

### 5.2 Grain Boundaries

The formation of transient trap states at GBs has been revealed by Prezhdo et al., which accounts for the picosecond-scale evolution of Σ5(210) GB of CsPbBr_3_.^107^ The MLP model was used to characterize the nanosecond MD trajectories, allowing rare events on long time scales to be captured. GB deformation occurs on ∼100 ps time scales and is accompanied by fluctuations between shallow and deep trap states; notably, deep traps are highly transient, typically persisting for only a few hundreds of femtoseconds, in contrast to the longer-lived shallow states. Another important insight is that localizations of electrons and holes at the GB can extend carrier lifetime by reducing their likelihood of simultaneous trapping and recombination. The evolution of these localized states was correlated with structural descriptors such as bond angles and Br–Br distances, with mutual information analysis indicating that variations in bond angles are more influential than Br–Br separation.

Independent work on the CsPbBr_3_ Σ5(120) GB has further shown that GB sliding strongly affects the dynamic evolution of deep trap states.^108^ Training a DP model followed by 1 ns of MD simulation, it was observed that within a few picoseconds the GB spontaneously slides, increasing the Pb–Pb distance from 3.54 Å to 4.34 Å, suppressing Pb–Pb hybridization, and increasing the band gap. Over the subsequent hundreds of picoseconds, thermal fluctuations and distortions shorten the Pb–Pb distance again, allowing deep LUMO states to re-enter the forbidden gap and form traps, thereby narrowing the HOMO–LUMO gap. Overall, the occurrence of trap states is strongly correlated with the average Pb–Pb distance and the coordination environment of Pb atoms.

To actively suppress GB trap states, the same group used a DP model to probe the effect of axial strain on the Σ5(120) GB in CsPbBr_3_ and its electronic structure.^109^ They considered tensile, strain-free, and compressive conditions. Tensile strain induces large-scale and slow structural fluctuation at the GB, which drives deep LUMO levels into the band gap and promotes trap formation (Figure 13). Only compressive strain effectively suppresses structural fluctuations and eliminates deep traps, ultimately driving the GB to reconstruct to an amorphous configuration. This reconstruction produces a nonmonotonic evolution of the band gap: partial recovery of the HOMO–LUMO gap is accompanied by strengthened Pb–Br coordination and suppressed Pb–Pb interactions, leading to a structurally and electronically stabilized GB.

**Figure 13:**
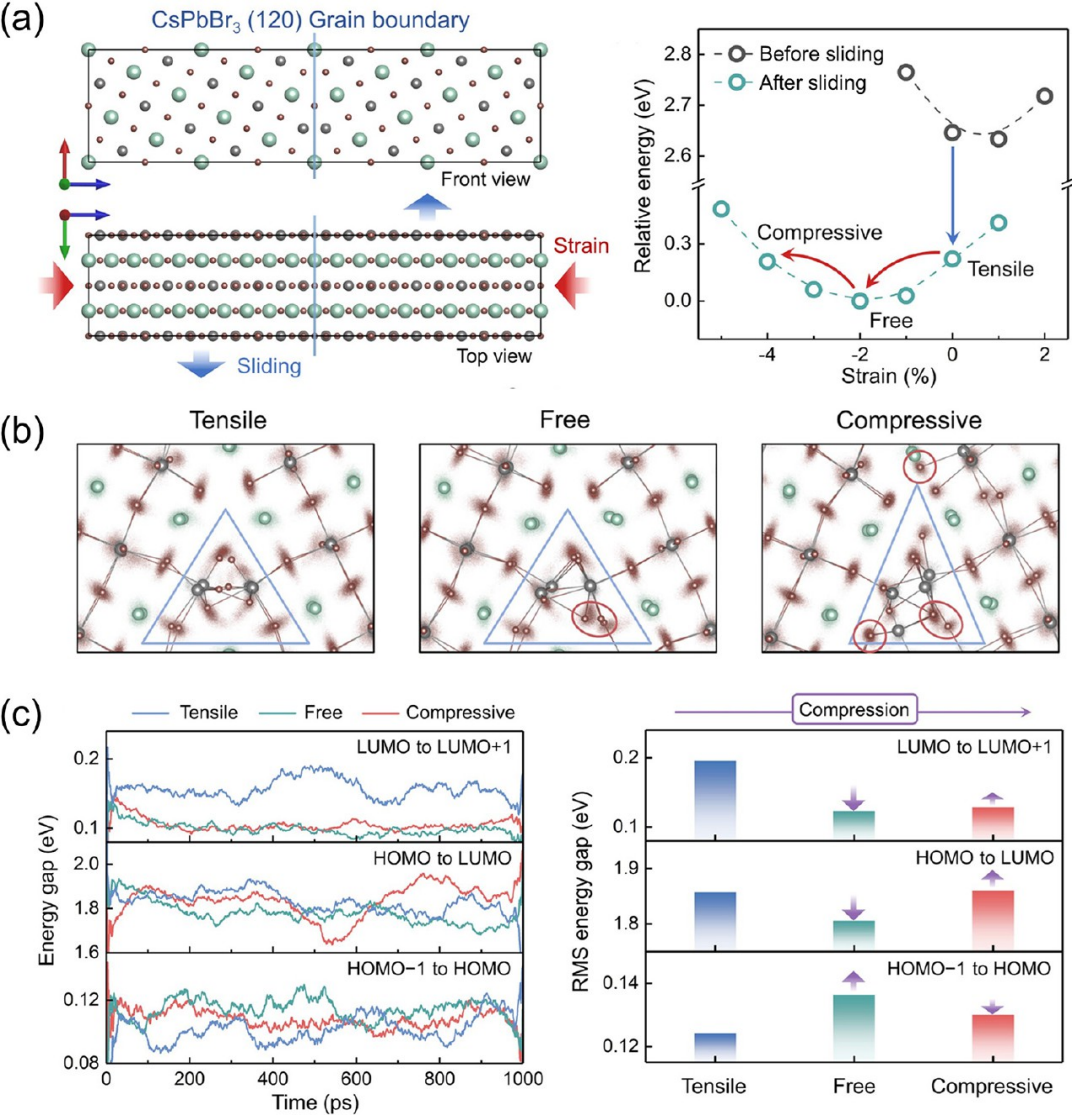
(a) Crystal structure of the CsPbBr_3_ GB model. (b) Structural oscillations of the GB models along the 1 ns MLMD trajectories. (c) Evolution of the energy gap of the GB models over the same MLMD trajectories. Adapted with permission from ref 109. Copyright 2024 American Chemical Society.

Alternative mitigation strategies were proposed by Wu et al., demonstrating a self-healing mechanism or self-passivation at Σ5(210) GB of CsPbBr_3_ under Br-rich conditions using a DP model.^110^ Focusing on Br interstitials (Br_i_), they showed that both isolated Br_i_ and the GB can introduce electron or hole trap states, but the Br_i_ diffusing toward the GB can break the Pb–Pb dimers and thereby remove the associated traps, effectively healing the GB. In the nanosecond-scale simulations, Br_i_ hops on time scales of tens to hundreds of picoseconds, temporarily creating trap states during migration. Overall, Br_i_-doped GBs remain trap-free for most of the time, display improved stability, enhance charge separation, and promote exciton dissociation.

Wu et al. also investigated Σ5(210) GBs doped with Br_i_ or Pb_i_ and used this self-passivation framework to rationalize experimentally observed long carrier lifetimes.^111^ An improved MLP, trained on over 10^5^ DFT configurations, including sufficient nonequilibrium structures from 100 to 1600 K, and coupled with NAMD, enabled a direct comparison of stoichiometric GBs, Br_i_-doped GBs, and Pb_i_-doped GBs. The results show that a pristine GB induces only ultrafast, transient trapping with minimal impact on the carrier lifetime τ. In contrast, Br_i_ passivates undercoordinated Pb sites, stabilizes the GB, and extends τ to ∼1.3 μs. Pb_i_, by comparison, initially creates shallow traps and, after GB sliding and structural rearrangement, generates deep midgap states that shorten τ from 1.18 to 0.72 μs. These studies illustrate how MLP-enabled, long-time-scale simulations can bridge the gap between experimental observations of GB-limited transport and a microscopic understanding of defect-driven trap formation, passivation, and reconstruction at perovskite interfaces. A summary of representative studies applying MLPs across these domains is provided in Table 1.

**Table 1:** Summary of Representative Studies Applying MLPs to Investigate Phase Stability, Defects, and Interfaces in Metal Halide Perovskites

category	system	features	DFT	MLP	training data set and source	refs
phase transition and stability	CsPbX_3_ (X = Cl, Br, I)	octahedral tilting	LDA, vdW-DF-cx, SCAN, PBE + D3	NEP	10.5281/zenodo.7454223	^85^
	CsPbI_3_	dynamic local structure, transient domains	LDA	ACE	10.5281/zenodo.8252919	^82^
	CsSnBr_3_, CsSnI_3_	pressure–temperature diagram, thermoelectric performance	PBE	NEP	800 configs	^87^
point defects	CsPbI_3_@surface and bulk defects	defect stability, instability temperature	PBE	DP	7.7 k configs	^101^
	CsPbBr_3_@V_Cs_	thermal conductivity, lattice distortion	PBE + D3	DP	5.6 k configs	^104^
ion migration	γ-CsPbI_3_ (pristine and doped)	dopant-induced relaxation, migration barrier	PBEsol	MACE	4 k configs	^112^
GBs	CsPbBr_3_ Σ5 (120) GB	ion migration on GBs, structural reconstruction	PBE + D3	DP	10.5281/zenodo.8138960	^98^
phase transition and stability	MAPbBr_3_, CsPbI_3_	octahedral tilting, molecular orientation	r^2^SCAN	GAP, ACE	10.5281/zenodo.7948045	^86^
	MA_1‑x_FA_*x*_PbI_3_ α, β, γ phases	morphotropic phase boundary (MPB), octahedral tilt patterns	SCAN + rVV10	NEP	10.5281/zenodo.14992798	^92^
GBs	CsPbBr_3_, Σ5(210) GB, stoichiometric, and with Br_i_ and Pb_i_	transient/persistent trap states at GBs, carrier lifetime	PBE + D3	DP	100 k configs	^111^
interfaces	FAPbI_3_, α-phase, various facets	surface terminations, moisture, HBs	SCAN + rVV10	DP	github.com/PolyUyini/FAPbI-water	^106^
phase transition and stability	PEA_2_Ma_n‑1_Pb_n_I_3n+1_ (PEA, PMA, BA, MA spacers)	layer-dependent octahedral tilting, distinct phase transitions	SCAN + rVV10	NEP	10.5281/zenodo.11120638	^96^

## 6 Perspective

The analysis in the preceding sections highlights a major shift in the computational modeling of inorganic and hybrid lead halide perovskites. The widespread use of MLPs now makes it clear that this approach has moved well beyond the proof-of-concept stage. MLPs have provided valuable insights into thermodynamic phase stability, ion migration pathways, and complex interfacial behavior that influence the optoelectronic performance of these materials. At the same time, the development and applications of MLPs for perovskite photovoltaics require careful oversight. ML models are intrinsically agnostic to the underlying physics; they infer the shape of the PES solely from the quantum mechanical data used for training. This flexibility is a key advantage over empirical potentials, but it also means that the learned PES remains limited by the quality, diversity, and coverage of the training database. Therefore, MLPs should not be treated as a simple black-box solution. In the context of perovskite research, important aspects of MLP efficiency and transferability still need further improvement and systematic assessment.

The first major challenge concerns the efficiency of current MLPs for perovskites. There remains a nontrivial trade-off between accuracy and computational cost. High-accuracy MLPs, especially those based on equivariant descriptors or message-passing architectures, are still relatively expensive. Their cost is orders of magnitude lower than those of DFT but remains much higher than that of traditional empirical potentials. This trade-off becomes even more severe when the training objective is extended to include additional observables that are critical for optoelectronic applications, such as partial charges, dipole moments, and polarizabilities. Incorporating these tensorial properties is essential for a realistic description of dielectric screening and charge-carrier dynamics in hybrid perovskites, but it further slows down the simulations. In practice, most high-fidelity MLP simulations are therefore limited to nanosecond time scales and system sizes of at most ∼10^5^ atoms. While this already represents a major advance compared to AIMD, it still leaves a substantial gap relative to experimental conditions. Device degradation, phase segregation, and ion migration often unfold on microsecond-to-second time scales and involve micron-scale morphological features. As a result, current MLP-based simulations struggle to capture the rare events that frequently initiate failure, which in turn limits their ability to provide truly predictive guidance for device design and long-term stability.

To help navigate the accuracy-efficiency trade-off in practice, we propose the following guidance for selecting MLP architecture: (i) For phenomena that require extensive phase-space exploration, long time scales (e.g., >10 ns), or very large supercells (e.g., >10,000 atoms), local descriptor-based models are currently the most practical option. Their superior computational efficiency allows for statistical sampling and convergence that are often prohibitive for more complex models. (ii) For problems dominated by complex local chemical bonding rearrangements, such as defect chemistry, defect migration with bond reorganization, and surface/interfacial reactions, MPNNs are often preferable. While more computationally demanding, their improved data efficiency and ability to capture many-body correlations can deliver the chemical accuracy needed to distinguish between competing reaction pathways. (iii) For screening large compositional spaces (e.g., mixed-halide/mixed-cation alloys), where training a specific model for each composition is impractical, pretrained universal potentials provide a strong starting point for rapid estimation or as foundational models that can be efficiently fine-tuned to targeted chemistries and environments.

A second critical methodological challenge is model transferability. In the broader materials science community, there has been rapid progress toward large atomic models trained on large and diverse data sets.^74–79,113–117^ However, these models typically contain a very large number of parameters and thus have slow inference speeds, making them poorly suited to the long trajectories and extensive sampling that are often required in MD simulations of perovskites. For halide perovskites, there is therefore an urgent need for a universal and lightweight MLP explicitly designed for their compositional space. Ideally, such a model would be applicable out-of-the-box to mixed-cation and mixed-halide systems, without the need for extensive, case-specific retraining.

Addressing the technical limitations of MLPs is essential, but the perovskite field itself also poses many open scientific questions that urgently require advanced MLPs. In particular, MLPs are well positioned to make progress in areas where traditional methods have struggled. Beyond methodological improvements, the first key scientific frontier is achieving an atomistic understanding of perovskite nucleation and crystal growth. High-quality films are typically produced by crystallization from polar organic solvents (such as DMF or DMSO) or by antisolvent engineering. These processes involve complex interactions between ionic precursors and solvent molecules. Although reactive force fields such as ReaxFF have proven effective for modeling solid-state interfaces and grain boundaries, extending them to solution-phase crystallization presents distinct challenges. The primary obstacle lies in accurately describing the competing coordination dynamics between halide ions and organic solvent molecules (e.g., DMF, DMSO) around the Pb center. Standard reactive potentials are rarely parametrized with sufficient specificity to reproduce the subtle energetic balance among Pb-solvent and halide-solvent interactions, which are critical for determining desolvation barriers and nucleation pathways. At the same time, DFT-based MD is typically too expensive to reach the system sizes and sampling needed to describe these processes. Training MLPs for such solid–liquid environments, however, introduces its own set of distinct challenges. Unlike bulk solids, precursor solutions exhibit high configurational entropy, requiring the model to learn a diverse range of dynamic solvation structures involving organic molecules and ionic complexes. Moreover, crystallization is controlled by delicate energy differences mediated by weak interactions, including hydrogen bonding and dispersion, often requiring high-level DFT functionals for reliable labeling. Finally, nucleation is inherently a rare event; capturing the relevant transition states and critical nuclei for robust training generally requires enhanced-sampling strategies beyond equilibrium MD. Despite these difficulties, MLPs trained for solvated perovskite precursors could significantly change this situation by resolving the structure of prenucleation clusters and elucidating the dynamics at the solid–liquid interface, providing an atomistic route toward rational control of crystallization kinetics and film morphology.

A second scientific frontier is the reversible yellow-black phase transition, which remains poorly understood in both experiments and simulations. This transition involves a large structural change from a corner-sharing, photoactive perovskite phase to an edge-sharing, nonperovskite phase. Current MLPs and classical force fields often cannot simultaneously capture the relative energetics of these different polymorphs and the high energy barriers between them. Developing MLPs that can accurately resolve this complex energy landscape is therefore crucial. Such models are needed to uncover the microscopic mechanisms of degradation and to guide the design of additives that kinetically stabilize the black phase.

A third scientific frontier is halide segregation in mixed-halide perovskites under illumination, a dynamic, nonequilibrium process that fundamentally limits the voltage of wide-bandgap tandem cells. It is well established that mixed halides segregate under light and reverse in the dark.^118–122^ However, simulations have so far provided only limited atomistic insight into the photoinduced driving forces behind this behavior. A key reason is that standard MLPs are trained on ground-state potential energy surfaces and do not, by construction, account for changes in the electronic structure under illumination. The development of excited-state MLPs that explicitly couple ionic dynamics to photoexcitation could elucidate more physical mechanisms. Such approaches could clarify the mechanisms that control reversibility and help to identify compositions and processing routes that suppress halide segregation.

In conclusion, further progress will depend on addressing several interrelated challenges: improving data efficiency, enhancing transferability, and making models more robust to extrapolated configurations. As the community increasingly integrates MLPs with high-throughput experiments and automated simulation workflows, these models are poised to become a central tool for accelerating the development of halide perovskites from promising laboratory systems to stable, commercially viable technologies.
